# EURO-PROBE – Manual segmentations of the prostate and intraprostatic urethra on T2-weighted MRI

**DOI:** 10.1038/s41597-026-07738-7

**Published:** 2026-06-30

**Authors:** William Holmlund, Attila Simkó, Kamilla Kalmár, Szilvia Tótin, Ádám Hodoniczki, András Négyessy, Benedek Danka-Csontai, Rebeka Horváth, Enikő Koós, Viktor Rogowski, Christian Jamtheim Gustafsson, Péter Palásti, Zsuzsanna Fejes, Tufve Nyholm

**Affiliations:** 1 Department of Diagnostics and Intervention, Umeå University, Umeå, Sweden; 2 Department of Radiology, Albert Szent-Györgyi Medical School, University of Szeged, Szeged, Hungary; 3 Department of Urology, Albert Szent-Györgyi Medical School, University of Szeged, Szeged, Hungary; 4 Radiation Physics, Department of Hematology, Oncology, and Radiation Physics, Skåne University Hospital, Lund, Sweden; 5 Department of Medical Radiation Physics, Lund University, Lund, Sweden; 6 Department of Translational Medicine, Medical Radiation Physics, Lund University, Malmö, Sweden

## Abstract

The EURO-PROBE dataset provides manually created segmentations of the prostate and intraprostatic urethra on axial T2-weighted MRI volumes, complementing the existing LUND-PROBE (LUND Prostate Radiotherapy Open Benchmarking and Evaluation) dataset for prostate radiotherapy research. Delineations were performed by delineators with varying levels of experience, including radiologists, radiology and urology residents, and medical students, which enables both inter-reader variability analysis and stratification by reader experience. Across all 467 cases in the LUND-PROBE dataset, at least one paired segmentation of the prostate and intraprostatic urethra was created for each case, yielding 1,227 paired segmentations in total. By expanding the LUND-PROBE dataset, EURO-PROBE supports the development of automated segmentation tools, including for the urethra, which is a highly relevant organ at risk in focal dose escalated prostate radiotherapy.

## Background & Summary

Prostate cancer is one of the most commonly diagnosed malignancies among men worldwide^1^, with radiotherapy as one of the main options for curative treatment. To improve tumour control, focal dose escalated regimens targeting sub-volumes of the prostate have been successfully introduced^2^. In this context, the intraprostatic urethra emerges as a critical organ-at-risk (OAR) due to its location within the prostate clinical target volume (CTV) and the strong correlation between dose delivered and radiation induced toxicity^3,4^. Preserving urethral integrity is therefore essential to minimize genitourinary side effects and maintain quality of life for the patient.

During the radiotherapy treatment planning process, manual delineations of anatomical structures, including both CTVs and OARs, are performed on a pixel-wise basis, requiring labour intensive manual work that is subject to inter-reader variability. In recent years, automatic commercial solutions have become increasingly integrated into clinical practice. However, no currently available solution supports segmentation of the intraprostatic urethra, and open annotated magnetic resonance imaging (MRI) resources remain limited. Nevertheless, recent work has provided open expert annotations of the prostatic zones and urethra on T2-weighted (T2w) MRI and demonstrated deep learning performance at the level of the inter-reader variability between two expert readers, highlighting the potential of such methods for this task^5,6^.

Datasets that include annotations from multiple delineators provide additional value for method development and evaluation, as they enable evaluation across readers and analysis of inter-reader variability. This is especially relevant for small or visually less distinct structures such as the intraprostatic urethra.

Recently, the LUND-PROBE (LUND Prostate Radiotherapy Open Benchmarking and Evaluation) dataset was introduced as an open-access resource to support reproducible research in prostate radiotherapy and machine learning development^7^. While the dataset provides high-quality T2w MRI imaging, synthetic CT and multi-expert segmentations of the prostate CTV and surrounding structures, it does not include segmentations of the intraprostatic urethra.

In this work, we present a complementary extension to the LUND-PROBE dataset by providing manually created segmentations of both the prostate and intraprostatic urethra across all available cases, which we name EURO-PROBE to reflect the multinational collaboration behind it. The segmentations were created by delineators with varying levels of experience, including radiologists, radiology residents, urology residents, and medical students. Most cases contain segmentations from multiple readers, enabling analysis of inter-reader variability and stratification of reader experience. This may also support uncertainty quantification and the development of methods that account for annotation variability.

This DataDescriptor outlines the structure and delineation setup of the EURO-PROBE dataset. By complementing the LUND-PROBE dataset, our contribution expands its utility and provides a wider foundation for data driven research in focal dose escalated prostate cancer radiotherapy.

## Methods

### Image Data

For all patients included in the LUND-PROBE dataset^8^, axial T2w MRIs were used for delineation. These images were acquired on a 3 T MRI scanner (GE Healthcare, Chicago, USA) and resampled to an in-plane resolution of either 0.4375 × 0.4375 mm or 0.4688 × 0.4688 mm, with a uniform slice thickness of 2.5 mm. The difference in in-plane resolution reflects two acquisition protocols, referred to as the *old* and *new* protocol, respectively.

**Fig. 1:**
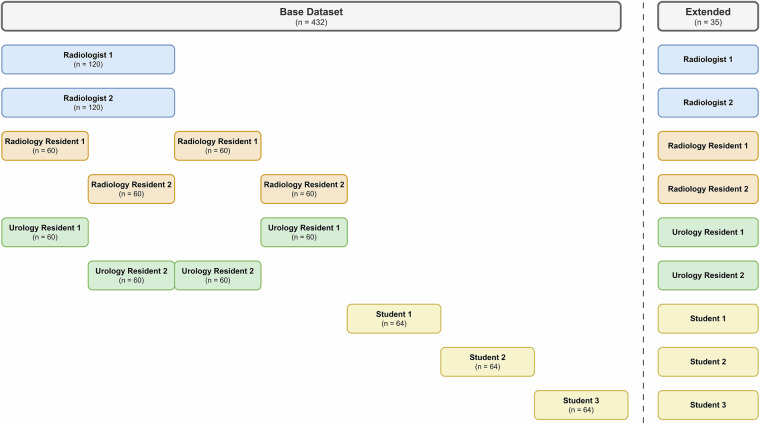
Data split. A schematic explanation of how the samples in both the base and extended part were split between the delineators.

The dataset consists of a base part (n = 432) and an extended part (n = 35). Approximately 25% of cases in the base part were acquired with the new protocol, whereas all cases in the extended part were acquired with the new protocol. For the base part, cases were randomly assigned to delineators following a structured overlap scheme to ensure multiple delineations for a subset of samples, see Fig. 1. In the extended part, all cases were independently delineated by all delineators. For each delineator, between 21.9% and 26.7% of their assigned cases in the base part were acquired with the new protocol, ensuring a balanced distribution of image quality across delineators.

### Segmentations

All delineations were performed manually, slice by slice, in the axial plane on T2w MRI volumes using 3D Slicer^9^. Delineators were instructed to separately delineate both the prostate and intraprostatic urethra. The prostate segmentation does not include the seminal vesicles. The intraprostatic urethra was delineated as a circular structure with a fixed 6 mm diameter in each slice, following the approach of Groen *et al*.^4^. The urethra was identified visually based on image contrast and anatomical cues, as no catheter was present on any image volume. The apex and external urethral sphincter were used to define the distal urethra, the verumontanum to guide the mid-prostatic segment, and the intraprostatic urethral angle was checked on sagittal reconstructions of the axial T2w MRI volume to account for curvature. Delineations performed in the axial plane were cross-checked on sagittal and coronal reconstructions of the same volume for continuity and manually adjusted when needed.

The delineator group consisted of two radiologists, each with three years of experience, two radiology residents and two urology residents, both groups with 1–2 years of experience, and three medical students who prior to the study received standardized training on prostate MR anatomy, imaging of prostate diseases, and the software application. For cases with multiple segmentations, each delineation was performed independently to enable unbiased comparisons between delineators. In the base part, no case contains multiple segmentations involving medical students.

## Data Records

The EURO-PROBE dataset is available at Zenodo^10^.

Segmentations are provided in the same compressed NIfTI format (.nii.gz) as the original LUND-PROBE data. This ensures that geometry information is preserved and that the files match the resolution and orientation of the corresponding axial T2-weighted MRI volumes.

Case naming convention follows that of the LUND-PROBE dataset. For each patient, the new segmentations are located in a subfolder named *euro_probe*. Within this folder, files are named according to the structure type and delineator, for example *mask_prostate_rad_1.nii.gz* and *mask_urethra_rad_1.nii.gz*, where *rad_1* indicates a segmentation from Radiologist 1.

To facilitate navigation of the dataset, a summary table listing the segmentations across all patients and delineators is provided with *segmentations.csv*. Each row corresponds to a unique patient identifier, and each column represents one delineator. An “X” indicates that a segmentation from that delineator is available for the corresponding patient.

## Technical Validation

Automated checks were performed to verify that the urethra remained continuous from its first to its last appearance in the image stack, with no intervening slices left empty. These checks also flagged cases with island voxels or holes, for both the prostate and urethra, and assessed whether the urethra maintained a consistent number of voxels across slices as a surrogate for shape continuity. Any deviations were flagged for manual review and correction.

**Fig. 2:**
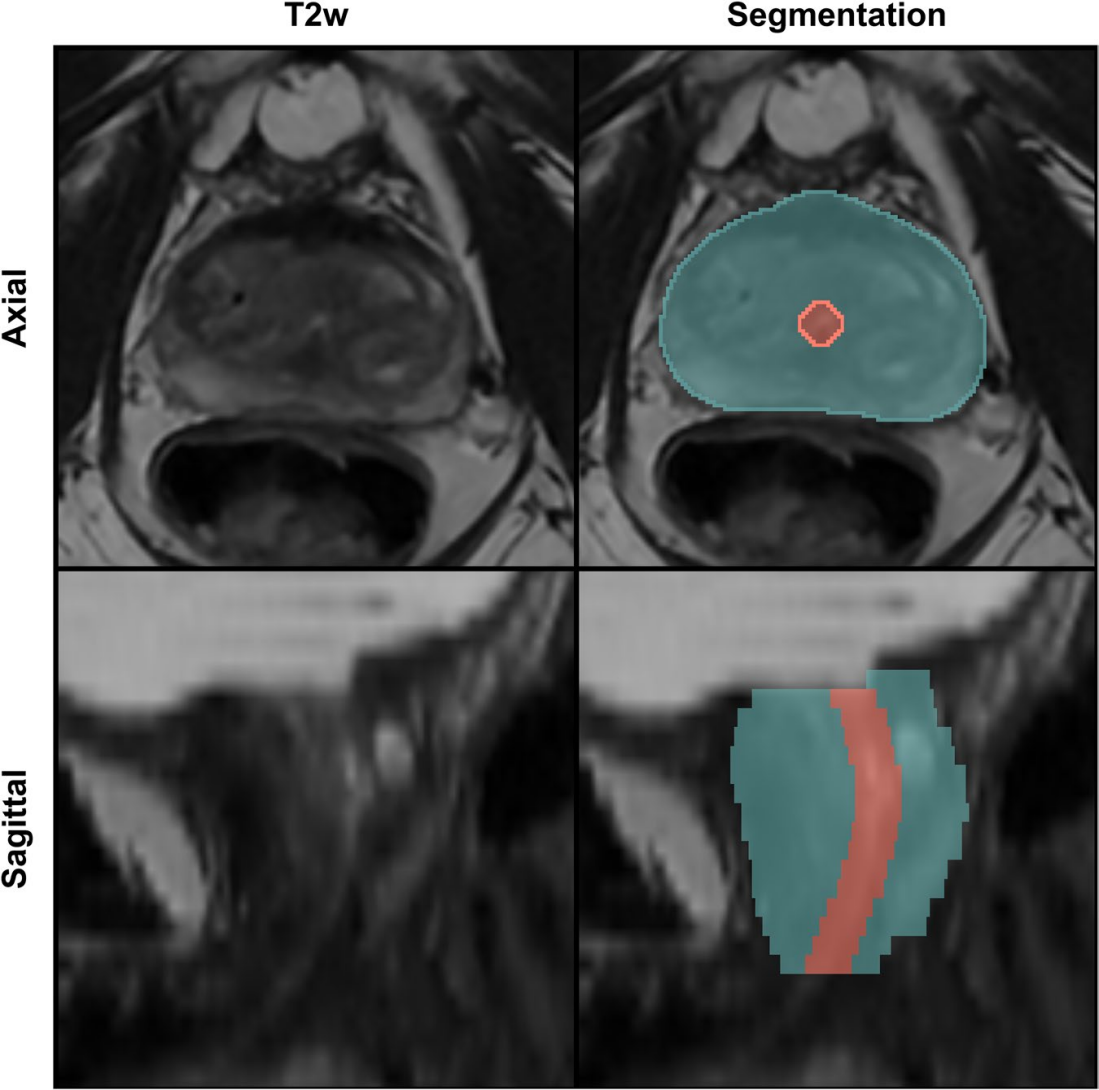
Example segmentation. An example segmentation of the prostate (light blue) and urethra (light red), displayed together with the corresponding axial T2-weighted image from the dataset.

A visual quality control step was performed to confirm that all segmentations were correctly aligned with the corresponding MRI volume. An example segmentation is provided in Fig. 2.

## Usage Notes

The EURO-PROBE extension of the LUND-PROBE dataset is ready for use without additional preprocessing. Its file structure and maintained spatial frame of reference is designed for straightforward integration with the original LUND-PROBE data, enabling a simple copy-and-paste of the data between the datasets.

### Ethics statement

EURO-PROBE is an annotation extension of the publicly available LUND-PROBE dataset and does not include newly collected patient data or new participant recruitment. The work involved manual segmentations generated on pre-existing de-identified imaging data obtained under the access conditions of LUND-PROBE through AIDA Data Hub^8^. No participant re-contact or re-identification was performed. Ethical approval, participant consent, and procedures for protection of participant information for the underlying clinical data are described in the original LUND-PROBE publication and repository documentation^7,8^.

## Data Availability

The EURO-PROBE dataset expands the LUND-PROBE dataset by providing complementary segmentations of the prostate and urethra for all cases. The EURO-PROBE segmentations are available at Zenodo^10^, and the LUND-PROBE dataset is accessible through the AIDA DataHub^8^.

## Code availability

Code for basic visualisation of the EURO-PROBE dataset follows the same principles provided in the LUND-PROBE GitHub repository (https://github.com/jamtheim/LUND-PROBE).

## References

[1] Bray, F. *et al*. Global cancer statistics 2022: GLOBOCAN estimates of incidence and mortality worldwide for 36 cancers in 185 countries. *CA Cancer J Clin* **74**, 229–263, 10.3322/caac.21834 (2024). 38572751 10.3322/caac.21834

[2] Kerkmeijer, L. G. W. *et al*. Focal Boost to the Intraprostatic Tumor in External Beam Radiotherapy for Patients With Localized Prostate Cancer: Results From the FLAME Randomized Phase III Trial. *J Clin Oncol* **39**, 787–796, 10.1200/JCO.20.02873 (2021). 33471548 10.1200/JCO.20.02873

[3] Leeman, J. E. *et al*. Radiation Dose to the Intraprostatic Urethra Correlates Strongly With Urinary Toxicity After Prostate Stereotactic Body Radiation Therapy: A Combined Analysis of 23 Prospective Clinical Trials. *Int J Radiat Oncol Biol Phys* **112**, 75–82, 10.1016/j.ijrobp.2021.06.037 (2022). 34711459 10.1016/j.ijrobp.2021.06.037

[4] Groen, V. H. *et al*. Urethral and bladder dose-effect relations for late genitourinary toxicity following external beam radiotherapy for prostate cancer in the FLAME trial. *Radiother Oncol* **167**, 127–132, 10.1016/j.radonc.2021.12.027 (2022). 34968470 10.1016/j.radonc.2021.12.027

[5] Holmlund, W. *et al*. ProstateZones – Segmentations of the prostatic zones and urethra for the PROSTATEx dataset. *Sci Data* **11**, 1097, 10.1038/s41597-024-03945-2 (2024). 39379407 10.1038/s41597-024-03945-2 PMC11461788

[6] Holmlund, W. *et al*. Automatic segmentation of the urethra and prostate zones with deep learning on T2-weighted magnetic resonance imaging. *Phys. Imaging Radiat. Oncol.* **38**, 100964, 10.1016/j.phro.2026.100964 (2026). 42011184 10.1016/j.phro.2026.100964 PMC13091743

[7] Rogowski, V. *et al*. LUND-PROBE - LUND Prostate Radiotherapy Open Benchmarking and Evaluation dataset. *Sci Data* **12**, 611, 10.1038/s41597-025-04954-5 (2025). 40216786 10.1038/s41597-025-04954-5 PMC11992069

[8] Rogowski, V. *et al*. LUND-PROBE - LUND Prostate Radiotherapy Open Benchmarking and Evaluation dataset. *AIDA Data Hub*10.23698/aida/lund-probe (2025). 10.1038/s41597-025-04954-5 PMC11992069 40216786

[9] Fedorov, A. *et al*. 3D Slicer as an image computing platform for the Quantitative Imaging Network. *Magn Reson Imaging* **30**, 1323–1341, 10.1016/j.mri.2012.05.001 (2012). 22770690 10.1016/j.mri.2012.05.001 PMC3466397

[10] Holmlund, W. *et al*. EURO-PROBE - Maunal segmentations of the prostate and intraprostatic urethra on T2-weighted MRI, *Zenodo*, 10.5281/zenodo.19109457 (2026). 10.1038/s41597-026-07738-7 PMC13323725 42380640

